# The Release of a Soluble Glycosylated Protein from Glycogen by Recombinant Lysosomal α-Glucosidase (rhGAA) In Vitro and Its Presence in Serum In Vivo

**DOI:** 10.3390/biom10121613

**Published:** 2020-11-29

**Authors:** Allen K. Murray

**Affiliations:** 1 HIBM Research Group, Inc., Chatsworth, CA 21053, USA; amurray1@glycantechnologies.com or allen@hibm.org; Tel.: +1-949-689-9664; 2 Glycan Technologies, Inc., P.O. Box 17993, Irvine, CA 92623, USA

**Keywords:** rhGAA, Pompe disease, glycogen, lysosomal α-glucosidase, GAA biomarker

## Abstract

In studies on the degradation of glycogen by rhGAA, a glycosylated protein core material was found which consists of about 5–6% of the total starting glycogen. There was an additional 25% of the glycogen unaccounted for based on glucose released. After incubation of glycogen with rhGAA until no more glucose was released, no other carbohydrate was detected on HPAEC-PAD. Several oligosaccharides are then detectable if the medium is first boiled in 0.1 N HCl or incubated with trypsin. It is present in serum either in an HCl extract or in a trypsin digest. The characteristics of the in vivo serum material are identical to the material in the in vitro incubation medium. One oligosaccharide cannot be further degraded by rhGAA, from the incubation medium as well as from serum co-elute on HPAEC-PAD. Several masked oligosaccharides in serum contain *m*-inositol, *e*-inositol, and sorbitol as the major carbohydrates. The presence of this glycosylated protein in serum is a fraction of glycogen that is degraded outside the lysosome and the cell. The glycosylated protein in the serum is not present in the serum of Pompe mice not on ERT, but it is present in the serum of Pompe disease patients who are on ERT, so it is a biomarker of GAA degradation of lysosomal glycogen.

## 1. Introduction

The deficiency of the lysosomal of α-glucosidase (GAA) in Pompe disease tissue, also known as type II glycogenosis and acid maltase deficiency, was identified in 1963 [1]. The initial publication did report the ability to release glucose from glycogen but did not specifically report α-1,6-glucosidase activity. The specificity of the lysosomal α-glucosidase to hydrolyze α-1,6 linkages was reported for the dog liver enzyme in 1964 [2] and its absence was reported in human Pompe disease tissues in 1970 [3]. The enzyme has multiple activities including α-1,4-glucosidase, α-1,6-glucosidase, transglucanase, transglucosylation, maltase and glucamylase. It is subject to substrate inhibition by maltooligosaccharides above a concentration of 5 mM [4,5,6,7,8]. However, the substrate concentration is not known in vivo or in vitro since the molecular weight or the concentration of the soluble fraction of glycogen is not known. The ability of the enzyme to degrade glycogen was reported as 91%, however, the inability to completely degrade Pompe glycogen when it was 80% degraded by the lysosomal α-glucosidase followed by phosphorylase and debrancher was reported in 1970 [5]. In another publication, the α-glucosidase was reported as able to convert 95% of the glycogen to glucose [6]. However, this is likely the comparison of glucose released by the enzyme compared to glucose released by acid hydrolysis as was previously published from the same laboratory. The ability to detect very small amounts of other carbohydrates was not available at that time. A problem with these two studies is that it appears that they used commercially isolated glycogen which I have found to be partially degraded by harsh isolation procedures. It is assumed that only glucose is released in vivo, but from earlier work with glycogen as a substrate, it appears that oligosaccharides are also released in vitro and are then degraded to glucose [9].

Enzyme replacement therapy using recombinant human GAA [rhGAA] for Pompe disease has been facilitated by the development of the knockout mouse model which is deficient in the lysosomal α-glucosidase [10]. There are reports of residual carbohydrate in the muscle tissue of the Pompe mouse following a course of enzyme replacement therapy (ERT) [10] as well as in biopsy muscle tissue from patients following ERT has also been reported [11,12]. The initial interest to begin this work was the presence of what was called residual glycogen in the cytoplasm of Pompe mouse tissue following ERT. At that time, I knew that glycogen was not a homopolymer of glucose, so the broader question was, could rhGAA completely degrade glycogen? It seems surprising that the question was not addressed before ERT began. This residual carbohydrate has been called glycogen in publications but it has not been isolated and identified. All that has been published is that it stains with PAS, indicating it contains carbohydrate.

The initial goal of this work was to determine if rhGAA could completely degrade glycogen. A core glycosylated protein, which is glycosylated primarily with inositol and sorbitol, iditol and has minor constituents of glucose, galactose, and mannose, as well as galactosamine and glucosamine, was identified. The mass of which consists of about 5–6% of the initial glycogen in the incubation tube [9]. It is likely that the residual carbohydrate material in tissue consists of the non-glucan portion of glycogen that cannot be degraded by rhGAA or possibly glycogen that has been modified by some pathophysiological process during storage. This present work is the result of an unexpected observation of that earlier work. The mass of glucose released by rhGAA and the residual glycosylated protein do not equal the mass of starting glycogen so about another 25% of the glycogen was unaccounted for. It is this unaccounted for glucan and a glycosylated protein containing primarily inositol and sorbitol which are the subjects of this report. About 70–75% of the mass of glycogen is released as glucose by the action of rhGAA in vitro. After approximately four days of in vitro incubation of glycogen with rhGAA, the glucose released reaches a plateau and no more glucose is released. No carbohydrate was detected in the medium that eluted after glucose by HPAEC-PAD on a CarboPac PA1 column. If the medium was first boiled in 0.1 N HCl for 30 min, a number of oligosaccharides were detected. Incubation with trypsin also exposed oligosaccharides for detection. It appears that this is a case of a protein masking carbohydrate which is unusual but some cases have been reported [13,14]. The soluble glycosylated protein in the medium is bound by Dowex 50W, which is evidence of binding as a charged entity such as a protein but it is not bound by concanavalin A which binds carbohydrates containing glucose or mannose, including glycogen [15]. Based on these characteristics and the possibility of the involvement of lysosomal exocytosis, serum was investigated and this soluble glycosylated protein was found to be present in serum.

## 2. Materials and Methods

### 2.1. Glycogen Substrates

Sigma, Type IX bovine liver glycogen, SigmaAldrich, St. Louis, MO, USA, is extracted by the method of Bell and Young, [16] which involves boiling and TCA precipitation of proteins at elevated temperature. This method is quite harsh compared to the method of isolation of the human glycogen in this report. All chemicals were of Reagent Grade or higher. Concanavalin A, monosaccharide and oligosaccharide standards and TFA were purchased from Sigma Aldrich, St. Louis, MO, USA. Dowex 50W was obtained from Bio-Rad, Hercules, CA, USA.

### 2.2. Human Glycogen Samples

Human glycogen samples were extracted by the method of Mordoh, Krisman, and Leloir [17] with the addition of five freeze-thaw steps to ensure the rupture of lysosomes. This method was chosen because it was reported that the isolated glycogen appeared to be identical to native glycogen isolated from liver as judged by its rate of sedimentation and its appearance under the electron microscope. Glycogen isolated by this method has been shown to be paracrystalline [18]. The glycogens were characterized for a number of parameters including average chain length, protein content, amino acid composition, RNA content, phosphate content, β-amolysis, iodine absorbance, interior chain length, and external chain length [19,20]. The protein content was less than one per cent for two of the three samples. All glycogens were hydrated for at least 18 h before incubations. Glycogen solutions were never frozen.

### 2.3. Source

Autopsy liver tissue from an 18-month-old female with Pompe disease (type II glycogenosis) and liver tissue from two adult male accident victims. The Pompe liver and the Control 1 liver were obtained at autopsy. In the case of Control 2, the patient was an organ donor on life support so the liver tissue was obtained immediately on termination of life support. All liver tissue was stored at −76 °C until the glycogen isolation. The case of the Pompe disease patient and an enzyme replacement trial with lysosomal α-glucosidase linked to low density lipoprotein has been previously reported [21].

The IRB approval was UC Irvine, UCI/ 2008-6631, and the genomic analysis of patients was reported [22].

### 2.4. Enzyme Assays

Recombinant human GAA (rhGAA) was provided by Sanofi Genzyme, Framingham, MA, USA, which is the 110 kDa precursor which is converted to the mature form in the tissue in ERT. Assay mixtures consisted of 1 mL volume containing 500 µg or more of glycogen as indicated, 50 mM sodium acetate buffer, pH 4.6, and 10 µL or 25 µL of rhGAA (5 µg/µL) as indicated. The reactions were incubated at 37 °C under toluene to prevent microbial growth. At various time points, as indicated in the figures, the reaction mixture was mixed on a vortex mixer, then centrifuged at 16,000× *g* for 5 min to precipitate any insoluble material. Then, a 100 µL or 200 µL aliquot was extracted and boiled for 5 min. The sample was then centrifuged at 16,000× *g* for 5 min to precipitate any insoluble material and the supernatant was analyzed for carbohydrates by HPAEC-PAD on a PA1 column. The remaining incubation mixture was mixed on a vortex mixer and returned to the water bath.

### 2.5. Carbohydrate Analysis

HPAEC-PAD was performed on a Dionex DX-600 ion chromatograph using a CarboPac PA1 column. (Thermo Fisher Scientific, Dionex, Thermo Elecdtron North America, LLC, Madison, WI, USA) The eluent was 150 mM sodium hydroxide, isocratic from 0 to 5 min, then a linear sodium acetate gradient from 5 to 25 min going from 0 to 57% 500 mM NaOAc in 150 mM NaOH at a flow rate of 1 mL/min. Fractions of 0.25 mL were collected using a Gilson 201 fraction collector. Fractions were partially reduced in volume on a Speed Vac to a volume less than 1.0 mL and then dialyzed overnight against 18.3 megohm water in 1.0 mL chambers against a 500 MWCO membrane. Fractions were taken to dryness in a Speed-Vac. The fractions were then hydrolyzed with 2 N TFA at 100 °C for two hours after which they were taken to dryness in a Speed-Vac. If it was determined that hydrolysis was incomplete, as evidenced by changes on passage through a Dowex column, samples were hydrolyzed again with 4 N TFA at 120 °C for 1 to 4 h. Monosaccharides and sugar alcohols were determined using a CarboPac MA1 column with isocratic elution with 480 mM NaOH at a flow rate of 0.4 mL/min. The waveform for carbohydrate analysis had a potential of +0.1 V from 0 to 0.40 s, −2.0 V from 0.41 to 0.42 s, +0.6 V from 0.43 to 0.44 s, and −0.1 V from 0.44 to 0.50 s with integration from 0.20 to 0.40 s. Data analysis was performed using Dionex Chromeleon 6.60 software.

### 2.6. Protein Determination

Protein determination was by a modification of the method of Lowry et al. [23]. A control experiment of protein determination on BSA showed no significant difference between samples of before and after hydrolysis for comparison.

## 3. Results

### 3.1. Incubation Medium Analysis

**Figure 1:**
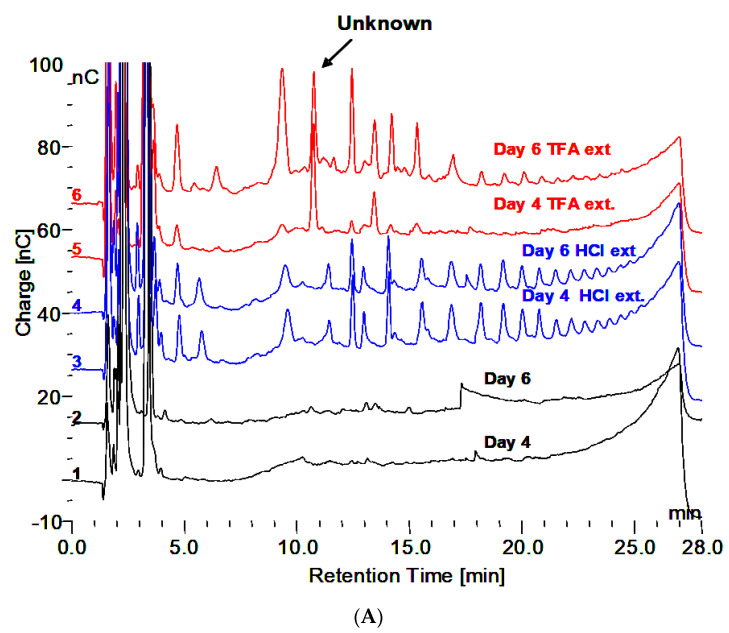

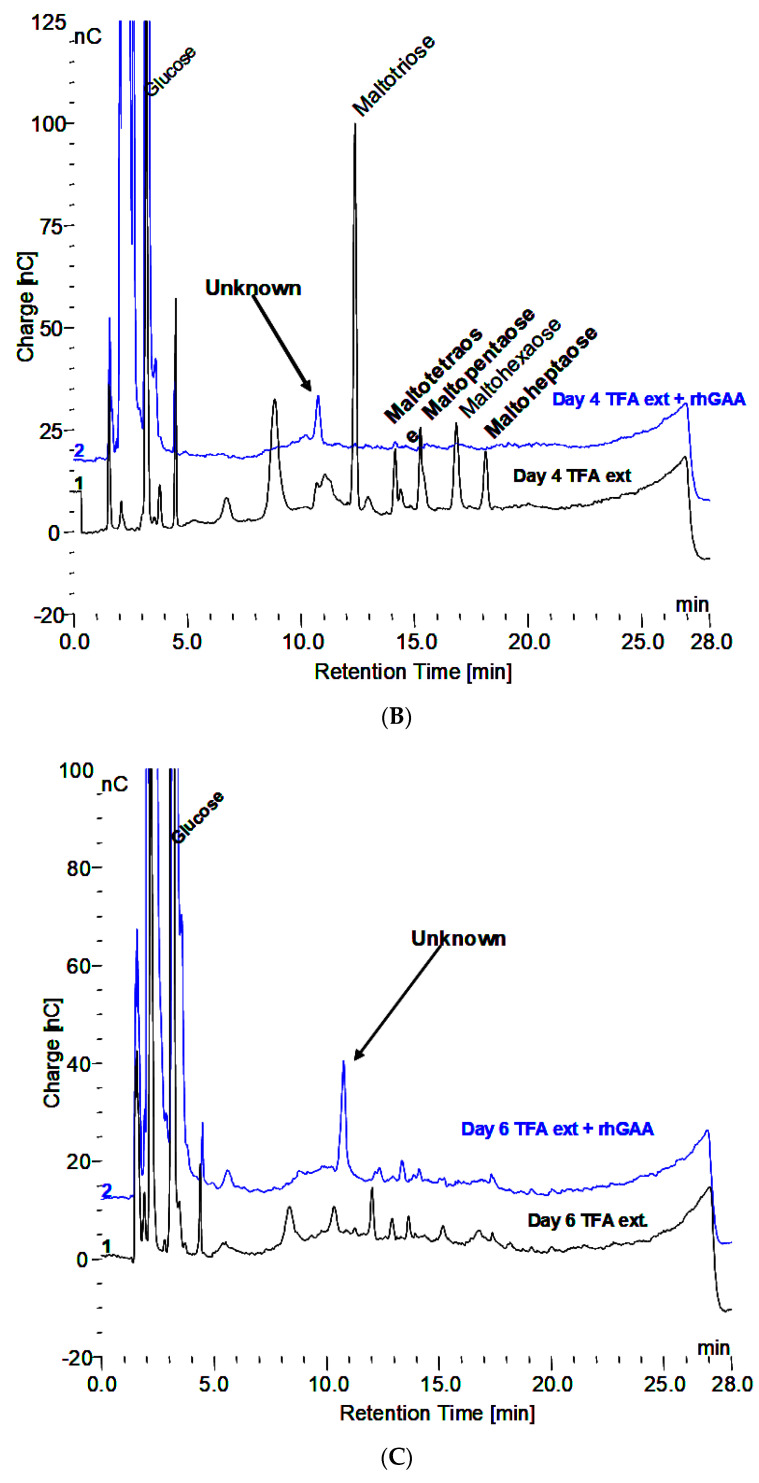
(**A**) Incubation medium of Control 2 with rhGAA after 4 and 6 days, HCl extract of the same and 2N TFA hydrolyzate of same. (**B**) Incubation medium of Control 2 glycogen with rhGAA after 4 days: 2N TFA hydrolyzate and same incubated with rhGAA. (**C**) Incubation medium of Control 2 glycogen with rhGAA after 6 days: 2N TFA hydrolyzate and same incubated with rhGAA.

Demonstration of the oligomers in glycogen and their relationship to degradation by rhGAA is shown in Figure 1A–C. Figure 1A shows the incubation medium from rhGAA degradation of Control 2 glycogen at 4 and 6 days of incubation, which only shows glucose released in the lower two plots, after four days no more glucose was released for up to 14 days. However, when the medium was extracted with 0.1 N HCl at 100 °C for 30 min, oligosaccharides were detected as shown in the middle two plots. Then, when the medium was treated with 2 N TFA for 2 h at 100 °C to degrade oligosaccharides to monosaccharides, the result was the surprising appearance of more oligosaccharides particularly with Control 2 glycogen in the 6 day medium as shown in the top two plots. It should be pointed out that at day 4, additional medium and enzyme were added. This particular glycogen sample, Control 2, appears to have a higher degree of complexity as shown in Murray [9]. This result was not as apparent in other glycogen samples. This particular glycogen sample was obtained from an organ donor who was maintained on life support until the organs could be harvested so this liver tissue was then frozen immediately. Some suggest that degradation of glycogen is apparent at 15 min after death. These results, and others, suggest that changes begin to take place very soon after death which may be why this glycogen may be somewhat different than other glycogens from tissue obtained at autopsy. Figure 1B,C demonstrates that the oligomers in TFA extracts can be degraded by rhGAA, however this is most apparent in the day 4 samples, which is due to the fact that the day 4 samples contains incubation medium from the beginning of the experiment. There are shifts in the retention times of residual peaks which appear to correspond to the residual material originally obtained from rhGAA degradation. The identification of the fraction which is released by the 0.1 N HCl contains about 18% of the mass of the initial glycogen sample. The characteristic which results in the appearance of the oligosaccharides on HCl extraction of the medium is of interest since this was totally unexpected. The residual material fractions all contain protein.

**Figure 2:**
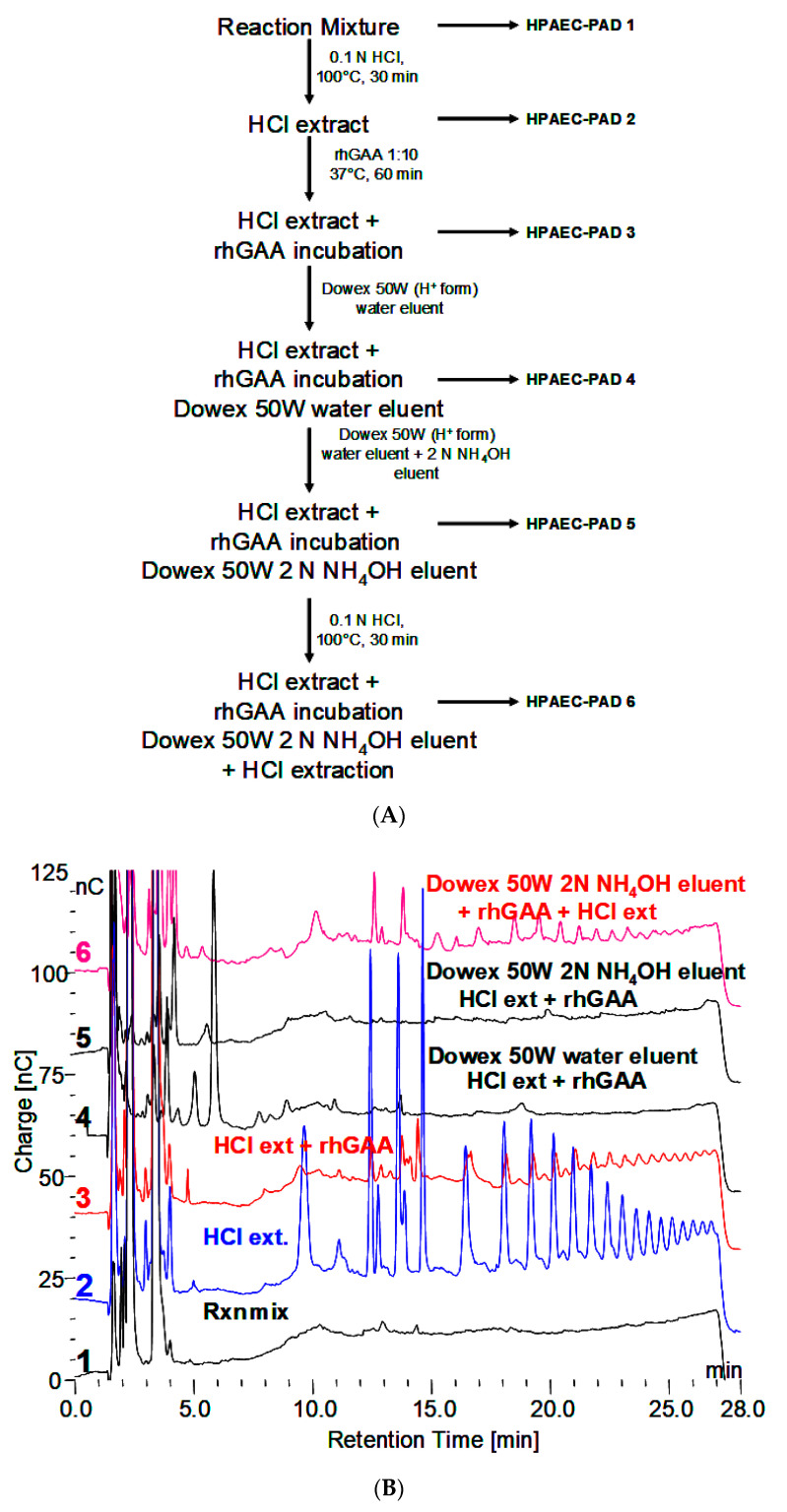
(**A**) Extraction procedures for incubation medium from rhGAA degradation of Control 2 glycogen after no more glucose is released. HPAEC-PAD numbers 1–6 refer to chromatograms 1–6 in 2B. (**B**) Results of samples analysis at each of sic steps in 2A.

Reaction mixture from rhGAA degradation of Control 2 glycogen was subjected to the scheme shown in Figure 2A. At each of the six steps, the sample was analyzed and the results are shown in Figure 2B.

### 3.2. Characteristics of the Glycogen Fraction that is not Degraded by rhGAA

1. HPAEC-PAD does not reveal any significant peaks that elute after monosaccharides. Which indicates no carbohydrates with ionizable hydroxyl groups are present?
2. Extraction with 0.1 N HCl at 100 °C for 30 min reveals maltooligosaccharides from DP 2 to about 18 on HPAEC-PAD.
3. The material in the incubation medium binds to a Dowex 50W ion exchange column and elutes in 2.0 N NH_4_OH. This is indicative of binding by a charged species such as protein or amino acids. After taken to dryness, it can be extracted with 0.1 N HCl at 100 °C to reveal the maltooligosaccharides.
4. Incubation with amyloglucosidase does not do anything to the samples.
5. Incubation with trypsin reveals some smaller oligosaccharides that elute in the region of up to about DP 4 and one at about DP 7 or 8. Additionally, trypsin treatment before HCl extraction appears to facilitate the appearance of more larger oligosaccharides. This is indicative of oligosaccharides being released or their appearance facilitated by the removal of protein.
6. Incubation with concanavalin A does not appear to bind the material. This indicates the absence of exposed glucose or mannose residues, including glycogen, which would be bound by the concanavalin A protein [14].

### 3.3. Summary of Characteristics

- Lack of chemical detection of ionizable hydroxyls of carbohydrate.
- Lack of biological recognition of carbohydrate by rhGAA, concanavalin A, or amyloglucosidase.
- Binding to Dowex 50W indicative of a charged species.
- Exposure of carbohydrate by incubation with a protease (trypsin).

These characteristics led to the conclusion that the material contains carbohydrate material which is masked by protein. There are reports in the literature of carbohydrate masked by protein. Since the material was not detected to be carbohydrate chemically, or by glycosidases and concanavalin A, it is possible that it is not recognized by the biological system. It was considered to be possible that it could be released outside the cell by the lysosomal exocytosis mechanism in which the lysosomal membrane fuses with the cell membrane and the lysosomal contents are expelled from the cell [24,25]. This has been shown for the export of stored glycogen from Pompe mouse cells in culture [26,27] and for the release of lysosomal enzymes in urine [28]. If that were the case, then it seemed reasonable that this material might be found in blood or urine. Normal human serum was investigated and the material was found to be present, indicating that this may be part of the normal mechanism of degradation for lysosomal glycogen.

### 3.4. Serum Investigation

**Figure 3:**
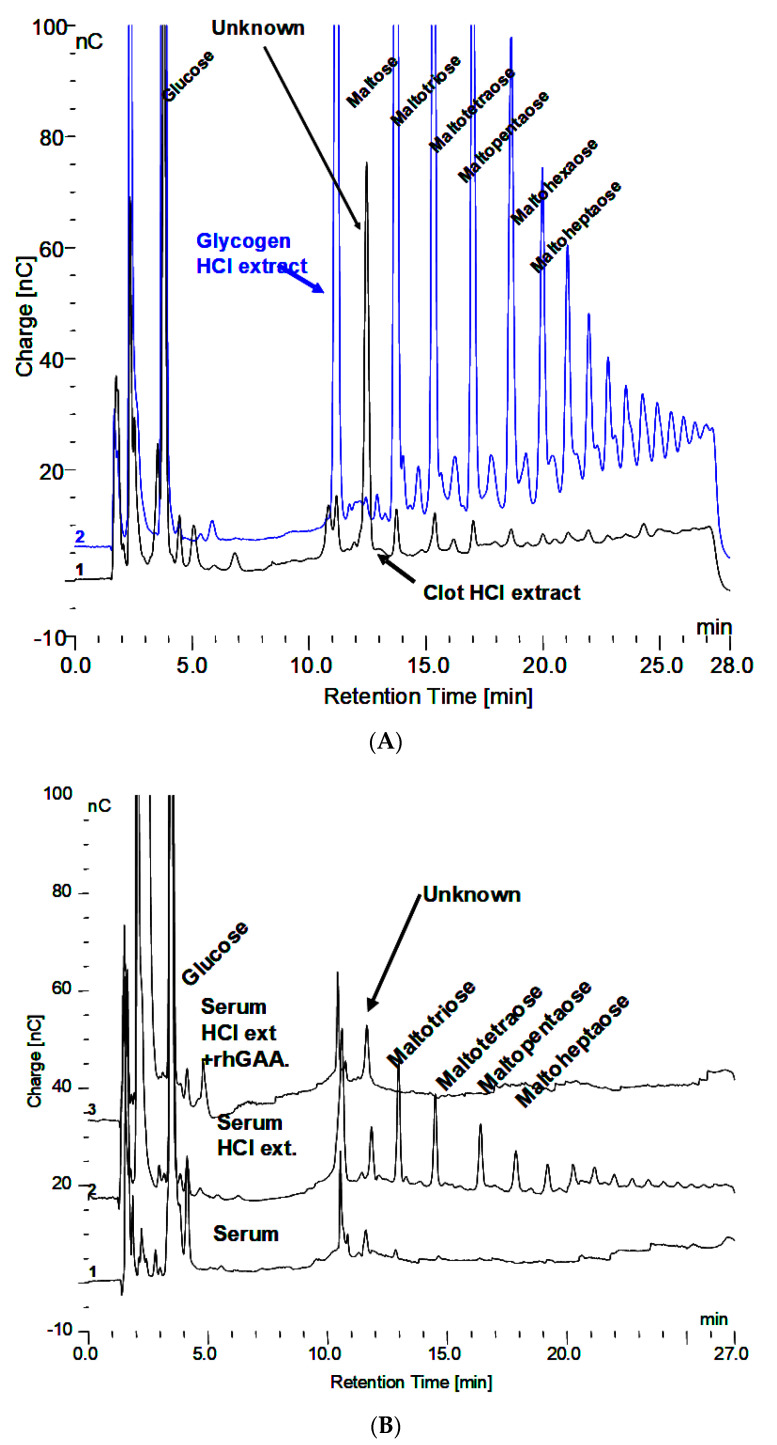
(**A**) Glycogen HCl extract showing maltooligosaccharides DP2-16 and clot HCl extract with array of maltooligosaccharides and the Unknown. (**B**) Serum, serum HCl extract, and serum HCl extract after rhGAA incubation.

About 200 µL of blood was obtained from a fingertip needle stick of a normal individual and added to 300 µL of 0.9% NaCl in a conical 1.5 mL tube and immediately centrifuged for 10 min at 10,000× *g* and allowed to clot. The serum was then diluted 1:10 and 1:20 and analyzed by HPAEC-PAD directly as well as after extraction with 0.1 N HCl for 30 min at 100 °C. The serum, HCl extract, and HCl extract following in vitro incubation of glycogen with rhGAA are shown in Figure 3A. The peak labeled “Unknown” is present in all of the serum samples analyzed. However, there was significant variability in the oligosaccharide content of the HCl extracts between different serum samples from the same source from day to day. The oligosaccharides varied but the unknown peak was consistently the same. The clot at the bottom of the serum but above the red blood cells was extracted. The HCl extract of the clot is characterized by abundant oligosaccharides. Since the clot contains fibrin and as a protein its function is to bind proteins or other components of blood, it is not surprising that the clot bound oligosaccharides, which appear to be conjugated to protein. Following HCl extraction, the oligosaccharides are readily degraded in vitro by rhGAA but the rhGAA does not degrade the unknown. The degradation of the oligosaccharides results in an increase in the size of the unknown peak.

**Figure 4:**
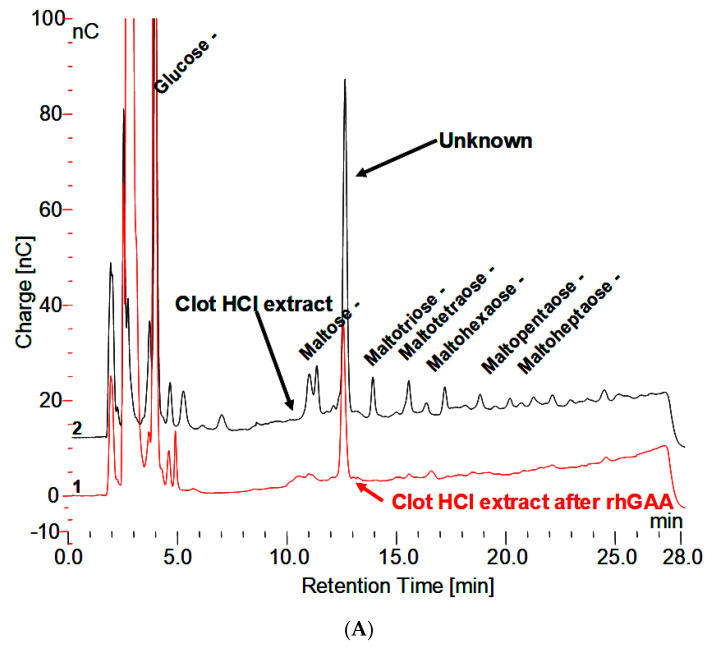

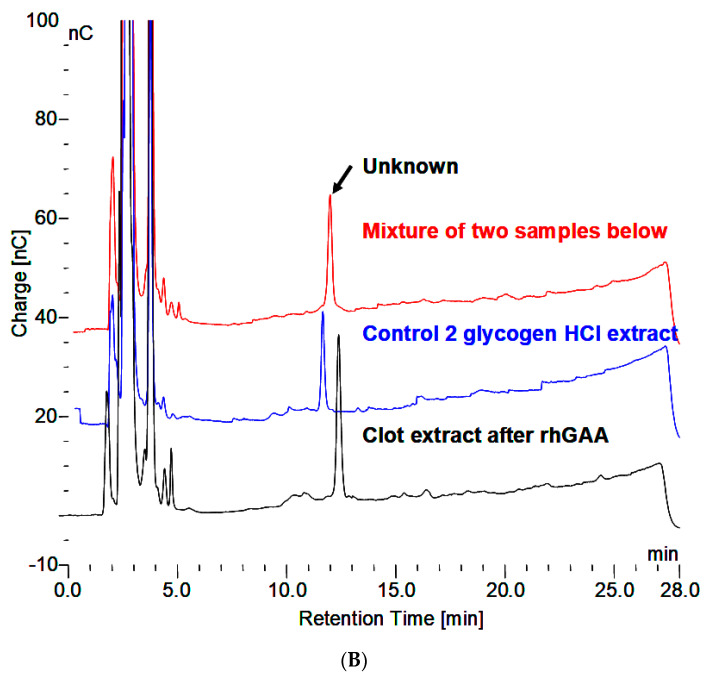
(**A**) Clot HCl extract before and after degradation with rhGAA demonstrating the Unknown is not degraded. (**B**) HCl extract of Control 2 glycogen, HCl extract of clot following rhGAA degradation, and a mixture of equal parts of both extracts demonstrating one symmetrical peak.

The HCl extract of the clot shown in Figure 3A contains almost the full array of malto-oligosaccharides from DP2-16. The small peaks between the oligosaccharide peaks in Figure 3A are the glycosylated protein peaks associated with the oligosaccharides [20], as well as the unknown. The rhGAA degradation of the oligosaccharides in the clot extract exposes the unknown and leaves the small peaks as well as some of the oligosaccharide peaks, which is shown in Figure 3B. The oligosaccharide peaks would likely not be there if a longer incubation was used. The chromatograms for the clot HCl extract before and after rhGAA degradation are shown in Figure 4A. The retention times for components can vary in different solutions as is the case with the HCl extract of incubation medium and the HCl extract of serum or the clot. This can be due to salt concentrations and other components of the solution. Therefore, to establish the identity of the material from the two different sources, equal volumes of both solutions containing the same amount of the unknown were combined and the mixture was chromatographed. In this case, the result was only one peak, which was symmetrical with no leading or trailing shoulders indicative of identity of the material from both sources as shown in Figure 4B. In this case, the Control 2 glycogen HCl extract was from day 8 of a rhGAA degradation of glycogen where the unknown was the only oligosaccharide remaining. It should be pointed out that there may be slight differences in absolute retention times of components but not in relative retention times. This is due to the fact that some chromatograms were obtained with only the electrochemical detector in use and other times with the addition of the photodiode array detector, which is in line ahead of the electrochemical detector, resulting in a slight delay in elution of peaks detected by the electrochemical detector.

### 3.5. Masking of Carbohydrate by Protein

**Figure 5:**
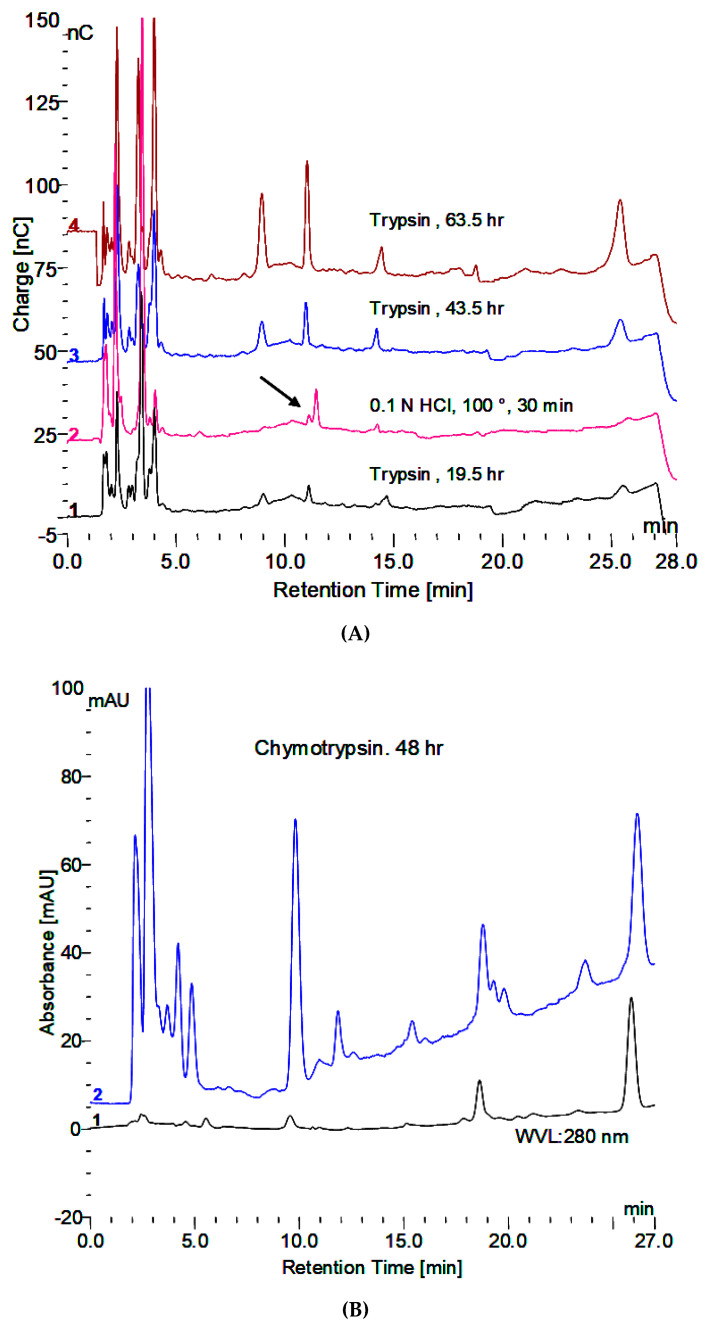
(**A**) Release of peptide bound oligosaccharides by trypsin and 0.1 N HCl. The HCl treatment was at the beginning. The trypsin treatment was for the indicated time at 37 °C under toluene. (**B**) Release of peptide bound oligosaccharides by chymotrypsin showing carbohydrate in blue and protein in black.

From the initial observation of the in vitro degradation of glycogen, that the apparent absence of oligosaccharides in the incubation medium could be overcome by boiling in 0.1 N HCl for 30 min or by trypsin, the concern became one of the comparison of the methods. An overnight or 24 h incubation with trypsin did not reveal as much of the terminal oligosaccharide, which is not degraded by GAA as was released by the HCl treatment. However, longer trypsin incubation releases much more of the material as shown in Figure 5A. It is also apparent that the HCl treatment results in a shift to a slightly longer retention time as well as variable release of the other oligosaccharides. The chymotrypsin treatment appears to release more oligosaccharide material, although it takes longer, as shown in Figure 5B. The products released are reproducible. The first two peaks with retention times of about 9 and 11 min as well as the last oligosaccharide with a retention time of 26 min are the major ones and as a result the ones of major interest. They all still have peptide material attached as shown in plots which show both the electrochemical detector and the absorbance at 280 nm. The retention times do shift slightly when other proteases are used due to the different specificity of which amino acids the proteases cleave.

### 3.6. Fraction Collection and Evidence of Protein Masking by Carbohydrate in Serum

The effect of doubling the concentration of trypsin used as well as chymotrypsin was tried. In 48 h incubation, doubling the trypsin concentration did not have a noticeable effect on the result. Chymotrypsin was more effective at the same concentration as trypsin. Proteinase K was also tried but there is a problem with proteinase K since it contains a number of oligosaccharides in the enzyme preparation which makes it problematic for collection of fractions.

**Figure 6:**
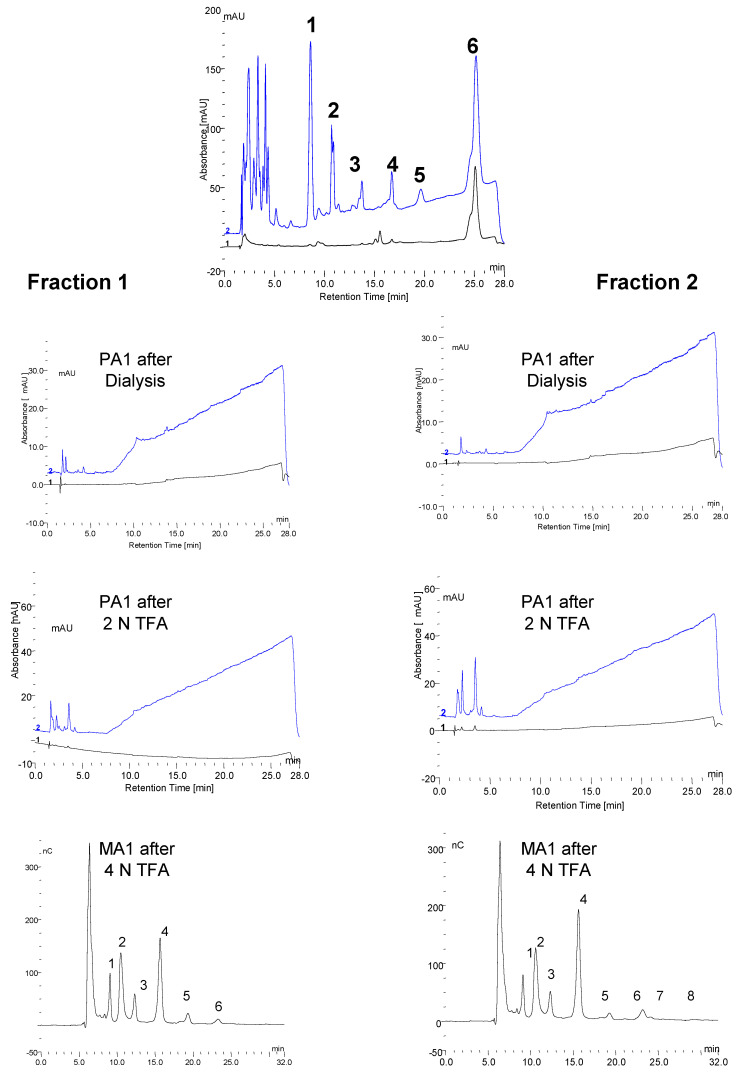
The top chromatogram shows the fractions collected. The three chromatograms on the left show fraction 1 after dialysis, after hydrolysis with 2 N TFA and after hydrolysis with 4 N TFA from top to bottom. The three chromatograms on the right show the same three treatments of Fraction 2. Carbohydrates: 1, *myo*-inositol:2, *epi*-inositol: 3, Xylitol: 4, Sorbitol: 5, Manitol: 6, Glucose: 7, Xylose: 8, Galactose.

**Figure 7:**
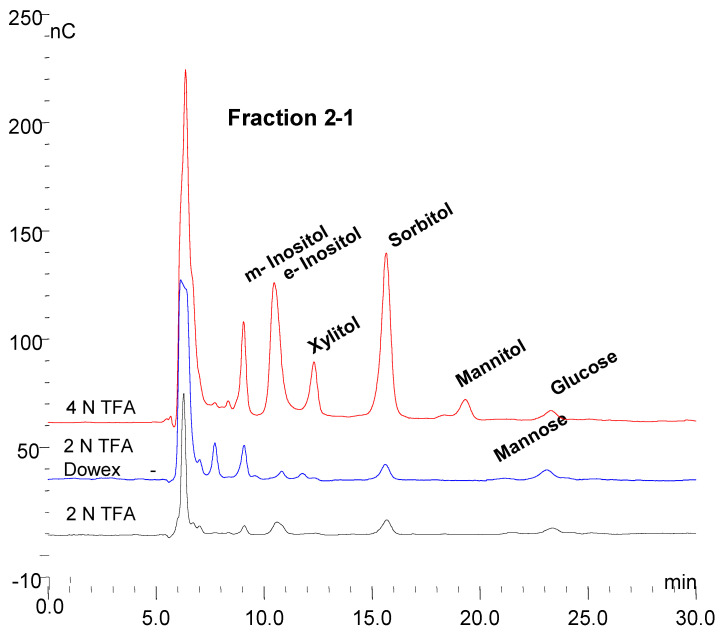
The monosaccharide chromatograms for Fraction 1 (2-1) are shown from bottom to top for 2 N TFA hydrolyzate, Dowex 50W column, and 4 N TFA hydrolyzate.

**Figure 8:**
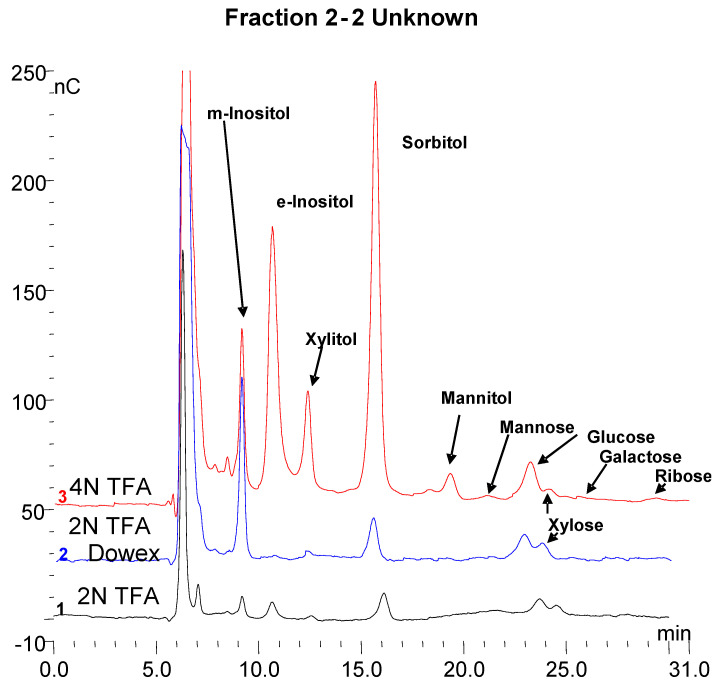
The monosaccharide chromatograms for Fraction 2 (2-2) are shown from bottom to top for 2 N TFA hydrolyzate, Dowex 50 column, and 4 N TFA hydrolyzate.

Six fractions were collected from a trypsin incubation mixture which are labeled 1–6 in the top panel of Figure 6. As mentioned earlier fractions 1, 2, and 6 are quantitatively the major ones of interest. These fractions were collected from multiple chromatographic runs into the same tubes. Below the top chromatograms, the parallel treatments of fractions 1 and 2 are shown. The fractions were partially concentrated on a Speed-Vac and then dialyzed against water at room temperature overnight using a 500 MWCO membrane in 1 mL dialyzers. Following dialysis, no oligosaccharides were detected as shown in the top chromatogram for each sample. There were a few small peaks at about 2–3 min retention time which represent sugar alcohols and a small peak at about 3.5 min which represents glucose galactose and mannose are not separated from glucose under these conditions. Next, the fractions were hydrolyzed in 2 N trifluoroacetic acid at 100 °C for two hours and then taken to dryness in the Speed-Vac. The fractions were then made up to 1.0 mL in water. Chromatography again on the PA1 column revealed increased monosaccharides but no oligosaccharides as shown in the second chromatogram for each fraction. The fractions were then analyzed by chromatography on an MA1 column which revealed inositol, sorbitol, hexoses, and xylose in 2-2 (Shown in Figure 7 and Figure 8). The samples were then passed over a Dowex 50W column to remove amino acids. The material which passed through was then analyzed by chromatography on the MA1 column which indicated increased inositol and sorbitol (shown in Figure 7 and Figure 8). This was likely due to additional hydrolysis on the resin which is not uncommon. Since it was apparent that everything had not been hydrolyzed the samples were then hydrolyzed in 4 N TFA at 120 °C for two hours, dried on a Speed-Vac and made up to 1.0 mL in water. Subsequent chromatography on an MA1 column revealed significantly more *m*-inositol, *e*-inositol, sorbitol, and xylitol (tentative) as well as hexoses and less xylose in 2-2 as shown in the bottom chromatogram for each fraction. During acid hydrolysis to release carbohydrates, although some carbohydrates are released with increasing time, others may be degraded. In this case, during the 4 N TFA hydrolysis, some glucose and a significant amount of xylose are degraded with increased time. This final TFA hydrolyzate is shown for both fractions in the bottom panel of Figure 6.

It is apparent from this sequence that the initial fractions from the collection in the 150 mM NaOH/NaOAc elution medium are altered by the dialysis to remove the salt. Those peaks are then not apparent when the dialyzed sample is chromatographed. However, TFA hydrolysis of the fractions demonstrates that the material was present but that it was masked. Therefore, it appears that the initial in vivo material from serum or the incubation medium from in vitro rhGAA degradation is masked by protein. After proteolysis with enzymes, there apparently is still enough peptide material attached to mask the carbohydrate after dialysis. It may be possible to remove more peptide material by using proteases with different specificities. It appears that after initial hydrolysis with 0.1 N HCl at 100 °C for 30 min to expose the carbohydrate, the removal of salt by dialysis then permits a configuration change to again mask the carbohydrate. This is the case for both the material from the in vitro rhGAA incubations and the in vivo material isolated from serum.

### 3.7. Monosaccharide Composition of Fractions

The three monosaccharide chromatograms for Fraction 1(2-1) after hydrolysis in 2 N TFA, followed by passage through a Dowex 50W column and hydrolysis in 4 N TFA are shown in Figure 7. After each step, the dried sample was made up to 1.0 mL. Since 25 µL samples were injected on the column in each case, the losses between steps were minimal. The increasingly broad injection peak is indicative of samples containing protein since proteins and amino acids are not retarded on the column.

**Figure 9:**
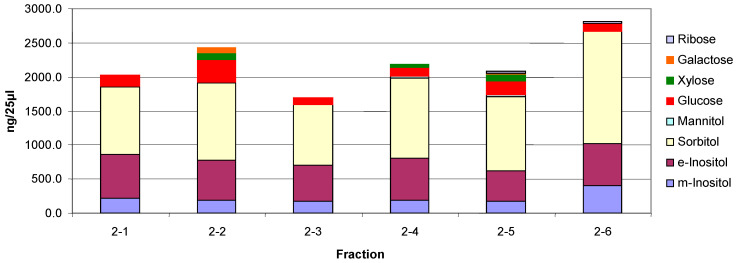
Monosaccharide composition of fractions.

The three monosaccharide chromatograms for Fraction 2(2-2) after hydrolysis in 2 N TFA, followed by passage through a Dowex 50W column and hydrolysis in 4 N TFA are shown in Figure 8. The monosaccharide composition of the six fractions is shown in Figure 9.

From the carbohydrate composition of the fractions it appears that the major components are the two inositols, sorbitol, xylitol, and mannitol are relatively similar and that the variability occurs in the monosaccharides. However, it is important to keep in mind that these are the monosaccharide compositions of oligosaccharides that still have some peptide attached. On incubation with rhGAA, all of them except 2-2 are degraded with an increase in 2-2 and free glucose, which indicates that although glucose is only a minor constituent, it likely is in a critical position. This is suggestive that at least a portion of the other glycopeptides are being converted to 2-2. There is still peptide attached but it is not known if the peptide is the same for all of them so it is not yet possible to determine with absolute specificity the quantitative interrelationships. It is very likely that there are multiple glycosylation sites, each having a different monosaccharide composition as will be discussed later.

The question of whether these in vivo fractions are intact components of glycogen or whether they have undergone some modification by GAA, or any other enzymes, is an open question since GAA does have glucanase, glucantransferase, and glucosyltransferase activities under the same conditions in which it has glucosyl hydrolase activity [4,5,6,7,8,9]. There is a commonly held belief that GAA only breaks glycogen down to glucose but it breaks down glycogen to some oligosaccharides which then are later degraded to glucose [9].

The carbohydrate composition of these soluble glycosylated proteins is unique by consisting mainly of inositols and sorbitol with some iditol. Inositol and sorbitol are not known to be found on any other protein. Literature searches do not reveal any glycosylated proteins published with these as the major carbohydrate. In fact, a search does not reveal any publication of a glycosylated protein with sorbitol

**Figure 10:**
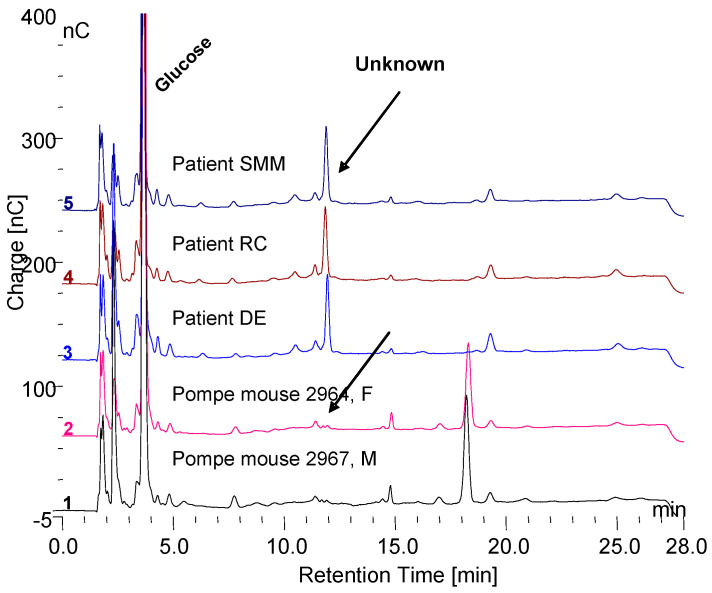
The 0.1 N HCl extracts of serum, 1:10 from two Pompe mice, age 8.7 months and three Pompe patients. The peak at about 18 min in the Pompe mouse serum is a glycosylated protein containing *m*-inositol, sorbitol and glucose as major components as well as galactose and mannose.

Figure 10 shows the HPAEC-PAD chromatograms of the 0.1 N HCl extracts from the serum of two Pompe mice that did not receiver ERT and the serum of three Pompe disease patients that are on ERT. These results are what would be expected if the unknown peak of interest (2-2 in Figure 6) is really a terminal degradation product of GAA degradation of glycogen. The unexpected result in Figure 10 is the large peak at about 18 min retention time in the serum of the Pompe mice. The retention time is what would be expected for maltoheptaose. The peak was collected and incubated with trypsin, which resulted in several peaks with shorter retention times. Each of those peaks was collected, hydrolyzed, and monosaccharide composition determined. Each had a different composition, but combined, they consisted of the inositols, iditol, and sorbitol with lesser amounts of glucose, galactose, and mannose. So, they appear to be related to the other glycosylated proteins from glycogen. The fact that this peak is present in the serum of Pompe mice not on ERT and absent from the serum of Pompe patients on ERT also implies that it is related. The serum of Pompe mice on ERT will be investigated as soon as it is available. It is unclear how this peak may be related to glycogen but based on its presence in the serum of Pompe mice not on ERT, its absence from normal serum or serum of Pompe patients on ERT and the similarity of its carbohydrate composition to the other soluble glycosylated proteins, it appears that it is related.

**Figure 11:**
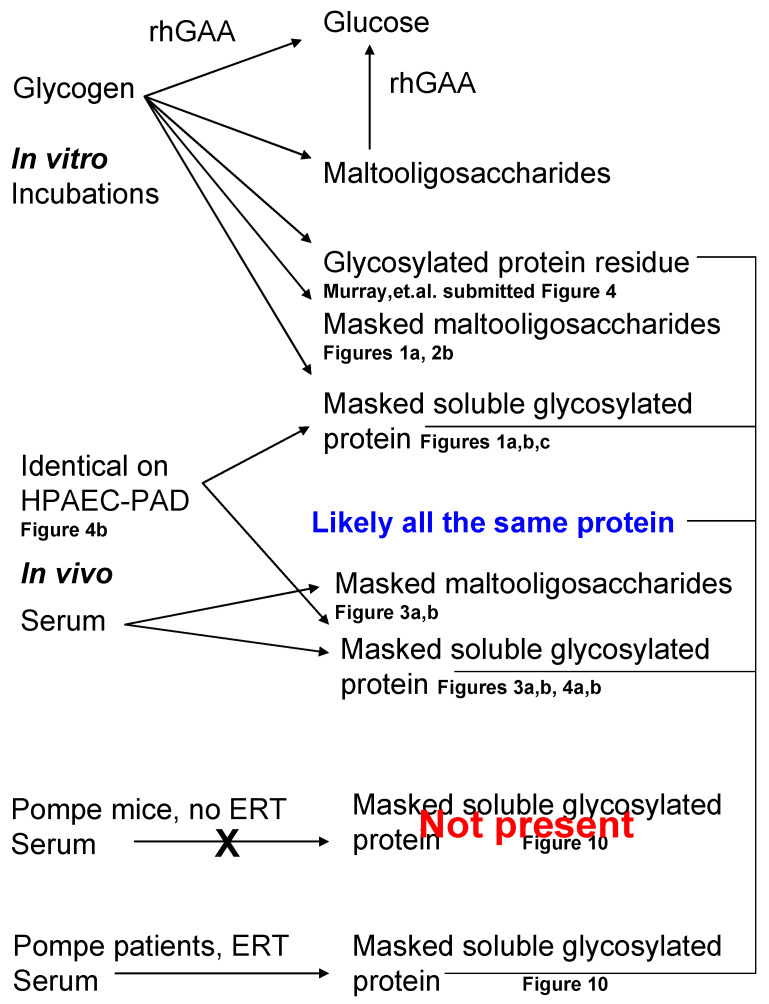
Summary of the metabolites discussed and reference to the figures showing them. The top portion shows metabolites from the in vitro incubations and the bottom portion shows in vivo metabolites isolated from serum.

A summary of the various fractions isolated following in vitro degradation of glycogen by rhGAA as well as the fractions isolated from normal serum, in vivo, is shown in Figure 11.

## 4. Discussion

A soluble fraction of glycogen that is not completely degraded in vitro by rhGAA contains at least 20% of the initial glycogen. The fact that the carbohydrate cannot be detected unless the incubation medium is boiled in 0.1 N HCl for 30 min or incubated with a protease such as trypsin or chymotrypsin for up to 48 h indicates that the carbohydrate is masked by protein. If the carbohydrate was exposed on the surface of the protein, it would be detected. Following protease incubation, all but one of the oligosaccharides can be degraded or modified in vitro by rhGAA. The one major oligosaccharide in serum that cannot be degraded in vitro by rhGAA following the HCl or protease treatment persists as a terminal metabolite of in vivo GAA degradation of glycogen which is peak 2-2 in Figure 9. As the peaks from serum that can be degraded in vitro by rhGAA disappear, peak 2-2 that is not be degraded increases in magnitude as the other peaks are degraded. As mentioned earlier, this has not been determined to be a quantitative conversion. This metabolite has an identical chromatographic retention time to an unknown previously detected from in vitro glycogen degradation by rhGAA and it has a similar monosaccharide composition as well.

Perhaps if calibrated with the proportion of glycogen degraded, this terminal metabolite could possibly be useful to quantitatively assess lysosomal glycogen degradation in vivo. However, since glycogen is not homogeneous, such a determination may be difficult to achieve without a better understanding of the structure of glycogen. It should be kept in mind that such experiments would not account for ongoing replenishment of lysosomal glycogen by phagocytosis which is assumed to be the case for turnover in lysosomal function in tissues and has been demonstrated in fibroblast culture [29]. One would assume that the lysosomal population would reflect a steady state appearance of glycogen uptake and degradation but the real time case may be more complex than that. Once more is known about the protein masking the carbohydrate, much more will be understood. It is possible an antibody could be raised against the protein or glycopeptides for analysis to determine the localization of complete lysosomal glycogen degradation.

It will be important to investigate the site of degradation of this glycosylated protein as it may be useful in the study of normal lysosomal glycogen in muscle. Liver or kidneys are likely sites of degradation. There is a neutral α-glucosidase in liver which is known to degrade maltooligosaccharides but it can not degrade glycogen [30]. The endogenous substrate for this enzyme is not known, yet there are other α-glucosidases present including some involved in glycosylation of glycoproteins. There is also a reported maltase in kidney for which there is no known substrate, but glucose is not a major component of this glycoprotein. It should be pointed out that glycogenin and the glycosylated protein appear to occupy different points of attachment to the polysaccharide.

Glycogenin is a self glucosylating protein which acquires 10–20 glucose residues, which then serve as the primer for the glucosyl residues of glycogen [31]. The glycosylated protein in this work is found associated with each oligosaccharide derived from the polysaccharide whether released by weak acid or enzymatic hydrolysis. Each one must differ in carbohydrate composition to account for their chromatographic separation; however, they could differ by a repeating unit. This implies that it likely is associated along the length of the polymer, while glycogenin is attached at the nonreducing end. It has been shown that glycogenin can attach xylose in the first position of glucosylation [32].

Comparison of the carbohydrate portions of unknowns 2-1 to 2-6 indicate that all have protein, inositols, sorbitol, mannose, glucose, and galactose and three appear to have xylose. The identification of xylitol is tentative.

During this work, the reasoning was that if the carbohydrate could not be detected by either chemical or biological means, then perhaps the cell could not recognize it as carbohydrate either. As mentioned earlier, there are precedents for the lysosomal exocytosis following degradation of a substance up to a point at which the lysosomal membrane fuses with the cell membrane and expels the material from the cell [24,25,26,27,28]. Although the terminal glycogen degradation product was found in serum, only a very small peak which was unresolved was found in urine that could possibly be attributed to this unknown metabolite. Based on co-chromatography, the soluble glycosylated protein found in serum in vivo which cannot be degraded by rhGAA in vitro appears to be the same as that in the incubation medium from rhGAA degradation of glycogen in vitro. Further degradation with proteases demonstrated that 6 oligosaccharide bound peptides were found, which could be expected since the HCl would not be a specific cleavage agent. On occasion, only four oligosaccharide-peptides were found. However, in all cases, there are quantitatively three major ones. All but one of the oligosaccharides can be degraded or at least significantly modified with rhGAA in vitro even though glucose is not a major constituent. Given the unknown effects of sometime different conditions or different proteases used, it is reasonable to suspect differences in the appearance of the minor oligosaccharide-peptide species. It should be pointed out that the extraction of the incubation medium of rhGAA degradation of glycogen in vitro also contains a series of masked maltooligosaccharides, which were not apparent before the HCl extraction, which then can be degraded by rhGAA as shown in Figure 1A,B, but they have to first be treated with HCl or protease to be detected. The same is true for the HCl extract from serum as shown in Figure 3B and Figure 4A. So, it is apparent that a significant portion of glycogen contains carbohydrate masked by protein which is degraded outside of the lysosome and subsequently the cell. Some of it is the typical maltooligosaccharide array which can be degraded in vitro by a further exposure to rhGAA and then there is the group of peptide bound oligosaccharides which contain inositol and sorbitol as their major carbohydrate components. It appears that the typical maltooligosaccharides, which are also masked, are peptide bound. However, based on other work, it would appear that all of the maltooligosaccharides contain the protein which is conjugated to inositol and sorbitol based on the chromatograms in which they appear to have the small peaks of the glycosylated protein [20]. The site(s) of degradation of the maltooligosaccharides masked by protein is also an important question because, although they can be degraded in vitro by rhGAA, they would not be exposed to GAA in vivo. If the protein were removed it, could be that they are degraded by one or more of the other α-glucosidases mentioned. It may be that the protein component of these protein bound maltooligosaccharides is the same as the soluble glycosylated protein unknown, fraction 2-2 in Figure 9 as suggested in Figure 11.

These results contribute to the observations of degradation of lysosomal glycogen. The question of where this soluble glycosylated protein is ultimately degraded is added to the list of unknowns as well as the site of degradation of the masked maltooligosaccharides. The fact that this soluble glycosylated protein has protein masking carbohydrate is significant. Such masking is known in biochemistry but I have not found any report for glycogen or for serum components [13,14]. To work out the details of this soluble glycosylated protein, sufficient quantities will be required which will require the collection of many fractions of the glycosylated protein associated with each oligosaccharide. The serum from 100–200 µL of blood contains as much soluble glycosylated protein as several 1 mL incubation tubes with 500 µg of glycogen in each. In comparison, the rat lysosomal fraction from about 140 g of muscle tissue was found to contain about 900 µg of glycogen in 10 mL. The original intent of this work was to isolate the lysosomal fraction from rat muscle and then monitor the degradation of the endogenous glycogen by the GAA in the fraction. However, if this work had begun with animals, the soluble glycosylated protein likely would not have been found or at least it would not have been found for a very long time. This now adds incentive to isolate the lysosomal glycogen and compare it to cytoplasmic glycogen as well as to do a comparison of proglycogen and macroglycogen from both the lysosomal fraction and the cytoplasmic fraction of tissue as well as to compare muscle and liver glycogen.

It is important to understand the masking mechanism to be able to quantify the soluble glycosylated protein as well as to understand the role of lysosomal glycogen in normal metabolism. Does this masking affect our methods of glycogen determination using amyloglucosidase? Should a proteolytic step be used before the amyloglucosidase degradation? These are now questions to be considered and experiments to be performed.

It is likely that the glycosylated protein residue from degradation of glycogen by rhGAA in vitro, the glycosylated protein associated with the masked oligosaccharides, as well as the glycosylated protein terminal glycogen degradation product are all the same protein based on their carbohydrate composition and possible location of their association with glycogen as indicated in Figure 11. It might be that degradation of the glucan then leaves protein attached and depending on ionic conditions, the protein may cover the carbohydrate to mask it.

One of the most interesting points of these results is that the glucose and sorbitol content of these polymers is greater than the quantity of free glucose in the same volume of blood. It is likely that one reason this has not been reported is due to the masking. There also appears to be some self-assembly of protein/carbohydrate when isolated fractions are dialyzed which also complicates things. This self-assembly is not unknown in polysaccharides. A TFA hydrolyzate of serum indicates that one of the most abundant carbohydrates is sorbitol, which is more abundant than glucose.

A similar glycosylated protein has been found in cellulose and corn starch, which also contains mannose, glucose, and galactose, with the major components being inositols, iditol, and sorbitol [20]. Sorbitol, also known as glucitol, is the intermediate between glucose and fructose. The inositol content is more difficult to determine since significantly more inositol is liberated by hydrolysis in 4 N TFA at 120 °C than is liberated in 2 N TFA at 100 °C, indicating that it is more difficult to extract. In fact, as shown in Figure 8, significantly more *m*-inositol was liberated by the passage of the sample over the Dowex 50W column of the 2 N TFA hydrolyzate and then much more by hydrolysis with 4 N TFA of the material passed over the Dowex 50W column. This is also true of *myo*-inositol in glycosylphosphotidyl inositol [GPI] anchor proteins. One reason that inositol and sorbitol may not have been reported as components of polysaccharides such as cellulose, starch, and glycogen may be that much of the earlier work was done using GLC where, for volatility, monosaccharides were converted to alditol acetates and *m*-inositol was added as an internal standard [33].

This terminal metabolite from the degradation of glycogen by GAA and its masking by protein will provide a mechanism as a biomarker by which to gain direct information on the function of lysosomal glycogen and perhaps its role in muscle physiology. Up to this point, our understanding of lysosomal glycogen has been limited to its accumulation in Pompe disease. Perhaps now we can begin to understand the role of lysosomal glycogen and if the impairment if its degradation pathway contributes to the pathophysiology of Pompe disease in a manner different than that attributed to the accumulation of glycogen. This information will permit a better basic understanding of Pompe disease and a better understanding of ERT and how it mimics the normal lysosomal glycogen pathway as well as how it may differ from that pathway if, in fact, it does differ.

This biomarker will enable an assessment of the effectiveness of ERT in Pompe patients in a relatively short time period of days. It could be monitored easily since only a pinprick is required for the small amount of blood needed. It will be possible to determine the most effective frequency of enzyme administration, particularly if it is found to vary among patients. The current two week interval may not be optimal for all patients so this will enable an assessment of GAA activity during the interval between administrations to assess the enzyme activity over the time period.

## 5. Conclusions

The breakdown of glycogen by rhGAA in vitro only releases enough glucose to account for about 75% of the mass of starting glycogen. About 5–6% is attributed to a glycosylated protein core which has inositols, sorbitol as the major carbohydrates, as well as glucosamine, galactosamine, glucose, and galactose. About another 18% is released as maltooligosaccharides masked by protein and the remainder is a soluble glycosylated protein also masked. The masked carbohydrate and the soluble glycosylated protein are found in normal serum, which is evidence that degradation of about 25% of the lysosomal glycogen occurs outside of the lysosome and outside of the cell. This appears to be facilitated by lysosomal exocytosis. The soluble glycosylated protein is a terminal degradation product of GAA degradation of lysosomal glycogen. This soluble glycosylated protein is not present in the serum of Pompe mice which are not on ERT but it is present in the serum of Pompe patients on ERT and normal individuals. This soluble glycosylated protein is a biomarker for degradation of glycogen by GAA and should be useful to assess ERT in patients. Since the glycosylated protein appears to be a terminal degradation product of GAA degradation of glycogen, it has potential to be very useful to monitor such degradation in relatively short time periods. As such, it should be possible to monitor the degradation of lysosomal glycogen essentially on a daily basis, if desired, to monitor such activity in the interval between administrations of rhGAA. This should enable a better understanding of the whole ERT process and may lead to improved treatment practices.

## Abbreviations

BSA	bovine serum albumin
ERT	enzyme replacement therapy
GAA	Lysosomal α-glucosidase
rhGAA	recombinant human lysosomal α-glucosidase
HPAEC-PAD	high performance anion exchange chromatography-pulsed amperometric detection
HPLC	high performance liquid chromatography
GLC	gas liquid chromatography
TFA	trifluoroacetic acid
PAS	periodic acid Schiff stain.

## References

[1] Hers H.G. (1963). α-Glucosidase deficiency in generalized glycogen-storage disease (Pompe’s disease). Biochem. J..

[2] Torres H.N., Olavarria J.M. (1964). Liver α-Glucosidases. J. Biol. Chem..

[3] Brown B.I., Brown D.H., Jeffrey P.L. (1970). Simultaneous Absence of α-1,4-Glucosidase and α-1,6-Glucosidase Activities (pH4) in Tissues of Children with Type II Glycogen Storage Disease. Biochemistry.

[4] Jeffrey P.L., Brown D.H., Brown B.I. (1970). Lysosomal α-glucosidase. I. Purification and properties of the rat liver enzyme. Biochemistry.

[5] Jeffrey P.L., Brown D.H., Brown B.I. (1970). Lysosomal α-glucosidase. II. Kinetics of action of the rat liver enzyme. Biochemistry.

[6] Palmer T.N. (1971). The substrate specificity of acid α-glucosidase from rabbit Mu muscle. Biochem. J..

[7] Palmer T.N. (1971). The Maltase, Glucoamylase and Transglucosylase Activities of Acid α-Glucosidase from Rabbit Muscle. Biochem. J..

[8] Hers H., Van Hoof F. (1966). Enzymes of glycogen degradation in biopsy material. Methods Enzymol..

[9] Murray A.K. The action of recombinant lysosomal α-glucosidase [rhGAA] and amyloglucosidase on bovine and human liver glycogen.

[10] Raben N., NFukuda O., Gilbert A.L., de Jong D., Thurberg B.L., Mattaliano R.J., Meikle P., Hopwood J.J., Nagashima K., Nagaraju K. (2005). Replacing Acid α-Glucosidase in Pompe Disease: Recombinant and Transgenic Enzymes are Equipotent, but Neither Completely Clears Glycogen from Type II Muscle Fibers. Mol. Ther..

[11] Del Rizzo M., Fanin M., Cerutti A., Cazzorla C., Milanesi O., Nascimbeni A.C., Angelini C., Giordano L., Bordugo A., Burlina A. (2010). Long-term follow-up results in enzyme replacement therapy for Pompe disease: A case report. J. Inherit. Metab. Dis..

[12] Van Der Ploeg A., Carlier P.G., Carlier R.-Y., Kissel J., Schoser B., Wenninger S., Pestronk A., Barohn R.J., Dimachkie M.M., Goker-Alpan O. (2016). Prospective exploratory muscle biopsy, imaging, and functional assessment in patients with late-onset Pompe disease treated with alglucosidase alfa: The EMBASSY Study. Mol. Genet. Metab..

[13] Dapson R.W. (1970). Histochemistry of mucus in the skin of the frog, Rana pipiens. Anat. Rec. Adv. Integr. Anat. Evol. Biol..

[14] Leger R.S., Cooper R., Charnley A. (1986). Cuticle-degrading enzymes of entomopathogenic fungi: Cuticle degradation in vitro by enzymes from entomopathogens. J. Invertebr. Pathol..

[15] Montgomery R. (1957). Determination of glycogen. Arch. Biochem. Biophys..

[16] Bell D.J., Young F.G. (1934). Observations on the Chemistry of Liver Glycogen. Biochem. J..

[17] Mordoh J., Krisman C.R., Leloir L.F. (1966). Further studies on high molecular weight liver glycogen. Arch. Biochem. Biophys..

[18] De Wulf H., Hers H.G., Piras R., Pontis H.G. (1972). Paracrystalline Glycogen. Biochemistry of the Glycosidic Linkage.

[19] Metzenberg A.B. (1980). Structural Feathers of Stored Glycogen in a Case of Pompe’s Disease [Glycogenosis Type II]. Master’s Thesis.

[20] Murray A.K., Metzenberg A.B., Nichols R.L. Polysaccharide similarities: Glycosylated protein cores of glycogen, starch and cellulose.

[21] Williams J.C., Murray A.K. (1980). Enzyme replacement in Pompe disease with an alpha-glucosidase-low density lipoprotein complex. Birth defects Orig. Artic. Ser..

[22] Alandy-Dy J., Wencel M., Hall K., Simon J., Chen Y., Valenti E., Yang J., Bali D., Lakatos A., Goyal N. (2019). Variable clinical features and genotype-phenotype correlations in 18 patients with late-onset Pompe disease. Ann. Transl. Med..

[23] Lowry O.H., Rosebrough N.J., Farr A.L., Randall R.J. (1951). Protein measurement with the Folin phenol reagent. J. Biol. Chem..

[24] Medina D.L., Fraldi A., Bouche V., Annunziata F., Mansueto G., Spampanato C., Puri C., Pignata A., Martina J.A., Sardiello M. (2011). Transcriptional Activation of Lysosomal Exocytosis Promotes Cellular Clearance. Dev. Cell.

[25] Rao S.K., Huynh C., Proux-Gillardeaux V., Galli T., Andrews N.W. (2004). Identification of SNAREs Involved in Synaptotagmin VII-regulated Lysosomal Exocytosis. J. Biol. Chem..

[26] Spampanato C., Feeney E., Li L., Cardone M., Lim J.-A., Zare F.A.H., Polishchuk R., Puertollano R., Parenti G., Ballabio A. (2013). Transcription factor EB [TFEB] is a new.therapeutic target for Pompe disease. EMBO Mol. Med..

[27] Li H.M., Feeney E., Li L., Zare H., Puertollano R., Raben N. (2013). WITHDRAWN: Clearance of lysosomal glycogen accumulation by Transcription factor EB (TFEB) in muscle cells from lysosomal alpha-glucosidase deficient mice. Biochem. Biophys. Res. Commun..

[28] Paigen K., Peterson J. (1978). Coordinacy of lysosomal enzyme excretion in human urine. J. Clin. Investig..

[29] Brown D.H., Waindle L.M., Brown B.I. (1978). The apparent activity in vivo of the lysosomal pathway of glycogen catabolism in cultured human skin fibroblasts from patients with type III glycogen storage disease. J. Biol. Chem..

[30] Brown B.I., Brown D.H. (1965). The subcellular distribution of enzymes in type II glycogenosis and the occurrence of an oligo-α-1,4-glucan glucohydrolase in human tissues. Biochim. Biophys. Acta.

[31] Roach P.J., DePaoli-Roach A.A., Hurley T.D., Tagliabracci V.S. (2012). Glycogen and its metabolism: Some new developments and old themes. Biochem. J..

[32] Rodén L., Ananth S., Campbell P., Manzella S., Meezan E. (1994). Xylosyl transfer to an endogenous renal acceptor. Purification of the transferase and the acceptor and their identification as glycogenin. J. Biol. Chem..

[33] Bukovac M.J., Olien W.C. (1982). Ethephon-Induced Gummosis in Sour Cherry (*Prunus cerasus* L.): 1. Effect on xylem function and shoot water status. Plant Physiol..

